# Masticatory Function in Elderly Individuals Living in Long‐Term Care Facilities in Brazil: Associations Between Objective and Subjective Measurements

**DOI:** 10.1111/joor.70133

**Published:** 2025-12-11

**Authors:** Lorena Tavares Gama, Mariana Marinho Davino de Medeiros, Yuri Wanderley Cavalcanti, Mario Augusto Brondani, Renata Cunha Matheus Rodrigues Garcia

**Affiliations:** ^1^ Department of Prosthodontics and Periodontology Piracicaba Dental School, University of Campinas São Paulo Brazil; ^2^ Department of Clinical and Social Dentistry Federal University of Paraíba João Pessoa Paraíba Brazil; ^3^ Department of Oral Health Sciences The University of British Columbia Vancouver British Columbia Canada

**Keywords:** aged, dental prosthesis, long‐term care, mastication, nursing homes, oral health

## Abstract

**Background:**

Although objective and subjective masticatory function measures may correlate, associated oral health–related factors may differ, highlighting the need for comprehensive assessment to support tailored care in long‐term care facilities (LTCFs).

**Objective:**

To investigate the association between objective and subjective masticatory function and the oral health status of older LTCF residents in Brazil.

**Materials and Methods:**

This cross‐sectional study included 187 residents (mean age = 78.7 ± 9.2 years) from nine LTCFs. Masticatory function was evaluated objectively (bi‐coloured chewing gum) and subjectively (‘Do you have trouble biting or chewing any kind of food?’). Oral health status was assessed by self‐perceived oral health, the number of natural teeth and posterior occluding pairs (POPs), xerostomia and dental prosthesis requirement. Data were analysed using multiple regression (*α* = 0.05).

**Results:**

Objective and subjective masticatory function were associated (*p* < 0.001). Older age (*p* = 0.006), low number of natural teeth (*p* = 0.001) and POPs (*p* = 0.004) and the dental prosthesis requirement (*p* = 0.016) were associated with poorer objective masticatory function. Poor self‐perceived oral health (*p* = 0.001), low number of POPs (*p* = 0.013), severe xerostomia symptoms (*p* = 0.001) and dental prosthesis requirement (*p* = 0.030) were associated with poor subjective masticatory function.

**Conclusion:**

Objective and subjective masticatory functions were associated and shared some common factors, number of POPs and the need for dental prostheses. However, objective measures (e.g., number of teeth) were linked to objective masticatory function, whereas self‐perceived factors (e.g., perceived oral health and xerostomia) were associated with subjective masticatory function.

## Graphical abstract

Objective and subjective masticatory functions were associated, but their related factors differed, highlighting the importance of considering both objective and self‐perceived indicators when assessing masticatory dysfunction in LTCF residents.

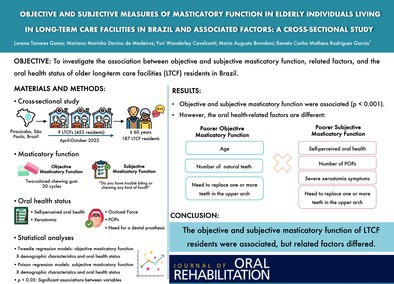

## 1 Introduction

Mastication is a physiological process and a determinant of health in older individuals. It plays a vital role in food processing, bolus formation, swallowing and nutrient intake [1, 2]. Limited masticatory ability can lead to a decline in general health, with a significant impact on older adults' quality of life [3, 4]. Beyond its nutritional consequences, reduced masticatory function has been associated with lower cognitive performance and represents a significant risk factor for dementia, mild cognitive impairment and increased mortality among older adults [5, 6]. This evidence highlights the relevance of mastication as an integral component of overall health in aging populations.

In long‐term care facilities (LTCFs), one in every three LTCF residents has difficulty chewing [7] and often experiences compromised oral health due to factors such as tooth loss, poor oral hygiene, and limited access to dental care [8]. Older people living in LTCFs have multiple missing teeth, replaced or not, and ill‐fitting dental prostheses that can further impair the ability to chew effectively [9, 10, 11] and negatively affect self‐perceived oral health [12]. Consequently, the comprehensive assessment of masticatory function in older adults, especially those living in LTCFs, is important.

Masticatory function can be assessed through both objective and subjective methods [13]. Objective measures yield quantitative data and typically involve evaluating food comminution or mixing ability using standardised test foods or materials to measure particle size reduction and the number of chewing cycles [13]. Among these, the mixing ability test analyzes how effectively two differently coloured materials are blended during mastication, serving as an indicator of oral motor coordination and efficiency [13, 14]. Although objective methods offer precise and reproducible results, they require specialised equipment and trained professionals [13], which may limit their applicability in clinical and institutional contexts.

Subjective assessment involves the administration of questionnaires to evaluate self‐ or proxy‐perceived masticatory function [13, 15]. These self‐reported measures capture individuals' perceptions and provide insights into how older people perceive their chewing ability and oral health, incorporating adaptive and psychological factors [16]. Such subjective measures are advantageous because they reflect the person's perspective on their masticatory function, which objective tests may not fully capture. They are particularly useful for older adults, including those living in LTCFs, as they are less demanding to administer and require minimal resources.

Although some researchers have reported correlations between objective and subjective measures of masticatory function [17], others have found no such association [18, 19], likely due to differences in study populations and/or methodologies. Both types of measure have been associated with common factors such as patients' prosthetic status [11], frailty [20], nutrition [21], and oral health‐related quality of life (OHRQoL) [22]. However, to date, no study has directly compared objective and subjective assessments of masticatory function and their associated oral health factors among residents of LTCFs. This gap in the literature limits our understanding of how clinical and functional variables interact to influence both objective performance and self‐perceived masticatory function and constrains the development of targeted interventions in institutional contexts.

Elucidating these associations may yield critical insights to inform strategies such as prosthetic rehabilitation aimed at restoring functional occlusion, nutritional monitoring to ensure adequate dietary intake despite oral limitations and structured oral health care planning to prevent further decline in oral status. Moreover, the present study expands the current evidence base by incorporating the institutional and socioeconomic context of Brazil, which differs from that of many countries where similar research has been conducted. Together, such approaches hold the potential to enhance masticatory function, promote better overall health outcomes and ultimately improve the quality of life of institutionalised older adults. Accordingly, this study investigated the association between objective and subjective measures of masticatory function and their related factors among older adults living in LTCFs in Brazil.

## 2 Materials and Methods

This study was approved by the Ethics Committee of the Piracicaba Dental School, University of Campinas (no. 66122917.6.3001.5418), and was conducted according to the principles of the Declaration of Helsinki. All participants provided written informed consent. The study has been reported following the STROBE guidelines [23].

This cross‐sectional study was conducted in LTCFs in Piracicaba, São Paulo, Brazil, in April–October 2022. All 15 LTCFs registered with the city council in 2022 were invited to participate; 6 declined. Thus, the study was conducted in 9 LTCFs that cared for a total of 452 residents.

We included LTCF residents aged ≥ 60 years and excluded those who were unable to respond to questionnaires due to cognitive disorders and/or hearing or communication problems. Cognitive disorders were evaluated using the Mini‐Mental State Examination, with a minimal cut‐off of 13 points used based on Brazilians' educational level [24].

A sample size estimation was performed based on the total number of older adults eligible to answer the research questionnaires (*n* = 213). Considering a 5% error, an effect design of 1.0, and an expected sample loss of 20%, the minimum required sample size was estimated at 172 participants. The final sample comprised 187 residents from nine LTCFs. After applying eligibility criteria for the regression analyses, 177 participants were included in the model of objective masticatory function and 176 in the model of subjective masticatory function, indicating that the study retained adequate statistical power to detect meaningful associations.

Through interviews and oral examinations, two trained researchers collected data on participants' (1) demographic characteristics, (2) objectively and subjectively measured masticatory function, and (3) oral health status (self‐perceived oral health, number of natural teeth, number of posterior occluding pairs [POPs], xerostomia and the need for a dental prosthesis). Sex was categorised as female or male.

### 2.1 Masticatory Function

Participants' masticatory performance was assessed objectively via the colorimetric analysis of bi‐coloured chewing gum (Vivident Fruit Swing Karpuz Asai Üzümü, Perfetti Van Melle, Turkey, Istanbul) [14]. Each participant was instructed to chew the gum for 20 masticatory cycles, based on several studies involving older adults [11, 14, 25]. The chewed gum was flattened to a 1‐mm thickness between two glass plates. The two sides were scanned, yielding JPEG files with a resolution of 300 dpi. The images were analysed using the ViewGum software (dHAL Software, Greece, Kifissia) to obtain variance of hue (VOH) values, which range from 0 to 1 and reflect the degree of colour mixing. Lower values reflect more mixing and thus better masticatory function.

Masticatory function was assessed subjectively using the question ‘Do you have trouble biting or chewing any kind of food, such as firm meat or apples?,’ adapted from the validated Brazilian version of the Geriatric Oral Health Assessment Index [6, 26]. Responses were dichotomized as ‘yes’ (indicating poor masticatory function) and ‘no’ (indicating good masticatory function) [6].

### 2.2 Oral Health Status

Participants' oral health status was assessed using two self‐reported variables (self‐perceived oral health and xerostomia) and three clinically assessed variables (number of natural teeth, number of POPs and the need for a dental prosthesis).

Participants were asked to rate their oral health on a Likert scale ranging from 1 to 5: ‘On a scale of 1 to 5, how do you rate your oral health?’. Higher ratings indicated better self‐perceived oral health [27].

Xerostomia was assessed using the Portuguese version of the Summated Xerostomia Inventory [28, 29]. Participants were asked to indicate how often they had the sensation of dry mouth and lips, and difficulty during feeding and swallowing. Response options were ‘never’ (1 point), ‘occasionally’ (2 points) and ‘frequently’ (3 points). Final scores range from 5 to 15, with higher scores reflecting more severe xerostomia symptoms.

The number of natural teeth was determined by counting the teeth in the oral cavity, excluding remaining roots and teeth indicated for dental extraction [30, 31]. The number of POPs (range, 0–8) was determined by counting natural, restored and prosthetic molars and premolars in contact during occlusion [32]. The need for a dental prosthesis in the upper and lower arches was assessed clinically and recorded as (1) the need for a complete denture, (2) the need to replace one or more teeth, or (3) none [33].

### 2.3 Statistical Analyses

Statistical analyses were performed using the SPSS software (version 20.0 for Windows; IBM Corporation, Armonk, NY, USA). Descriptive data analysis was conducted. As the VOH values were continuous, non‐normally distributed and centred around zero, adjusted Tweedie regression models were used to evaluate associations among objective masticatory function, demographic characteristics and oral health status. Factors associated with subjective masticatory function were identified using adjusted Poisson regression models. Variables with *p* < 0.20 in the crude model were included in models adjusted using the backward Wald procedure (i.e., regressive stepwise elimination of variables). The association between objective and subjective masticatory function was examined by Tweedie regression. Associations were interpreted using the regression coefficient (*B*), prevalence ratio (PR) and 95% confidence interval (CI). In the adjusted models, *p* < 0.05 was taken to indicate significant associations between variables. Multicollinearity was assessed for all variables in both adjusted regression models using the Variance Inflation Factor (VIF), with values below the threshold for potential concern (VIF > 5) and below the critical level (VIF > 10).

## 3 Results

**FIGURE 1:**
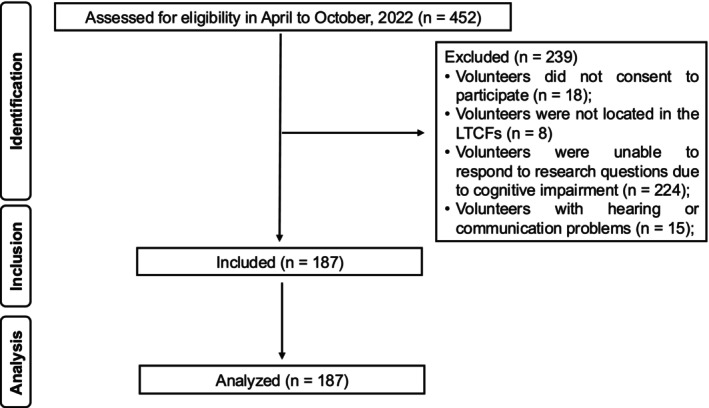
Flow diagram of participants.

Of the 452 residents of the 9 LTCFs, 18 did not consent to participate, 8 were absent from the facilities during the study period due to hospitalisation or other factors, and 239 were unable to respond to study questions due to cognitive impairment (*n* = 224) and hearing or communication problems (*n* = 15). Thus, 187 LTCF residents participated in the study (Figure 1).

The final sample had a mean (±standard deviation) age of 78.71 ± 9.18 years and a predominance of female participants (58.3% [*n* = 109]; Table 1). The mean objective masticatory function score was 0.66 ± 0.22, and 85 (45.5%) individuals reported poor subjective masticatory function. The mean self‐perceived oral health score was 3.66 ± 1.35, the mean number of natural teeth was 4.18 ± 7.51, and the mean number of POPs was 2.64 ± 3.26. Additionally, 116 (62.0%) participants required complete dentures in the upper arch and 108 (57.8%) participants required complete dentures in the lower arch.

**TABLE 1:** Participant characteristics.

Participant characteristics	
Objective masticatory function (mean ± SD)	
VOH	0.658 (±0.221)
Subjective masticatory function [*N* (%)]	
Yes	85 (45.5%)
No	102 (54.5%)
Gender [*N* (%)]	
Female	109 (58.3%)
Male	78 (41.7%)
Age (mean ± SD)	78.71 (±9.178)
Self‐perceived oral health (mean ± SD)	3.66 (±1.346)
Number of natural teeth (mean ± SD)	4.18 (±7.51)
POPs (mean ± SD)	2.64 (±3.256)
Xerostomia (mean ± SD)	7.78 (3.02)
Need for upper dental prosthesis [*N* (%)]	
Required a complete denture	116 (62.3%)
Needed to replace one or more tooth	31 (16.7%)
Did not need a dental prosthesis	39 (21.0%)
Need for lower dental prosthesis [*N* (%)]	
Required a complete denture	108 (58.0%)
Needed to replace one or more tooth	55 (29.6%)
Did not need a dental prosthesis	23 (12.4%)

Abbreviations: POP, posterior occluding pairs; SD, standard deviation; VOH, variance of hue.

The participant age, number of natural teeth, number of POPs and need for dental prostheses in the upper and lower arches were included in the adjusted Tweedie regression model (Table 2). This model indicated that the age (*B* = 1.01, 95% CI = 1.01–1.01, *p* = 0.006), number of natural teeth (*B* = 0.97, 95% CI = 0.96–0.98, *p* = 0.001), number of POPs (*B* = 0.971, 95% CI = 0.960–0.982, *p* = 0.004) and need to replace one or more teeth in the upper arch (*B* = 1.26, 95% CI = 1.13–1.41, *p* = 0.016) were associated with higher VOH values (i.e., poorer masticatory performance; Table 2).

**TABLE 2:** Variables associated with masticatory performance (VOH values), determined by Tweedie regression analysis (*n* = 176).

Variables	Crude Model				Adjusted model			
	*B*	*p*	95% Confidence interval		*B*	*p*	95% Confidence interval	
			Lower	Upper			Lower	Upper
Age	1.006	0.010	1.001	1.011	1.006	0.006*	1.002	1.011
Gender								
Female	1.010	0.863	0.901	1.132				
Male	Ref.							
Self‐perceived oral health	0.989	0.479	0.958	1.020				
Number of natural teeth	0.971	0.001	0.960	0.983	0.971	0.001*	0.960	0.982
POPs	0.971	0.005	0.952	0.991	0.972	0.004*	0.953	0.991
Xerostomia	1.006	0.478	0.989	1.024				
Need for upper dental prosthesis								
Required a complete denture	1.078	0.326	0.928	1.251	1.078	0.306	0.933	1.246
Needed to replace one or more tooth	1.297	0.017	1.047	1.607	1.298	0.016*	1.050	1.605
Did not need a dental prosthesis	Ref.				Ref.			
Need for lower dental prosthesis								
Required a complete denture	0.824	0.116	0.647	1.049	0.826	0.098	0.659	1.036
Needed to replace one or more tooth	0.847	0.204	0.656	1.094	0.859	0.245	0.666	1.109
Did not need a dental prosthesis	Ref.				Ref.			

Abbreviations: *B*, regression coefficient; POPs, posterior occluding pairs.

* *p* < 0.05.

The participant age, self‐perceived oral health, number of natural teeth, number of POPs, xerostomia and need for a dental prosthesis in the upper arch were included in the adjusted Poisson regression model (Table 3). This model indicated that poor subjective masticatory function was significantly associated with poorer self‐perceived oral health (*B* = 1.07, 95% CI = 1.03–1.11, *p* = 0.001), the number of POPs (*B* = 1.02, 95% CI = 1.01–1.03, *p* = 0.013), more severe xerostomia symptoms (*B* = 0.97, 95% CI = 0.96–0.99; *p* = 0.001) and the need to replace one or more teeth in the upper arch (PR = 0.85, 95% CI = 0.73–0.99, *p* = 0.030, Table 3). Furthermore, objective and subjective masticatory function were associated (PR = 1.19, 95% CI = 1.08–1.30; *p* < 0.001).

**TABLE 3:** Variables associated with subjective masticatory function, determined by Poisson regression analysis (*n* = 177).

Variables	Crude model				Adjusted model			
	*B*	*p*	95% Confidence interval		*B*	*p*	95% Confidence interval	
			Lower	Upper			Lower	Upper
Age	0.996	0.173	0.991	1.002	0.997	0.192	0.992	1.002
Gender								
Female	1.028	0.571	0.935	1.130				
Male	Ref.							
Self‐perceived oral health	1.071	0.001	1.032	1.110	1.070	0.001*	1.031	1.109
Number of natural teeth	1.006	0.112	0.999	1.012	1.006	0.053	1.000	1.012
POPs	1.014	0.042	1.001	1.029	1.016	0.013*	1.003	1.029
Xerostomia	0.972	0.001	0.956	0.987	0.973	0.001*	0.957	0.989
Need for upper dental prosthesis								
Required a complete denture	0.967	0.524	0.870	1.075	0.957	0.411	0.861	1.063
Needed to replace one or more tooth	0.860	0.056	0.736	1.004	0.848	0.030*	0.731	0.984
Did not need a dental prosthesis	Ref.				Ref.			
Need for lower dental prosthesis								
Required a complete denture	0.967	0.674	0.829	1.129				
Needed to replace one or more tooth	0.975	0.975	0.851	1.117				
Did not need a dental prosthesis	Ref.							

Abbreviations: *B*, regression coefficient; POPs, posterior occluding pairs.

* *p* < 0.05.

## 4 Discussion

This cross‐sectional study investigated the association between objective and subjective masticatory function and the oral health status of older people in LTCFs in Brazil, as well as associated factors. The findings demonstrated a significant relationship between objective and subjective measures of masticatory function. Moreover, the study identified several factors associated with masticatory function, highlighting the multifactorial nature of this condition in institutionalised older people. In addition, the results also revealed differences in the factors related to objective and subjective assessments.

Our findings, similar to those of Nedeljković et al. [17], showed that the objective and subjective masticatory functions of LTCF residents were associated, but that the factors associated with each differed. Poorer objective masticatory function was associated with age, number of natural teeth, number of POPs and need for a dental prosthesis in the upper arch. Poor subjective masticatory function was associated with poor self‐perceived oral health, number of POPs, xerostomia and the need for a dental prosthesis in the upper arch. Contrary to Limpuangthip et al. [19], who favoured objective measures of denture wearers' mastication, and in line with the recommendation of Pedroni‐Pereira et al. [18], our findings emphasise the need to use both clinical and self‐perceived measures when possible to monitor the masticatory function of older adults residing in LTCFs. Although more demanding, this combined approach may provide a more comprehensive understanding of residents' chewing ability and help to identify and address factors potentially influencing it.

The observed association between masticatory performance and age among older people is consistent with previous findings [16, 34]. Although tooth loss is not inevitable with advancing age, it is common among older people in Brazil [35] and affects chewing and swallowing [7]. Additionally, a recent cross‐sectional study showed that the masticatory performance of German community‐dwelling centenarians (aged 100–106 years) was worse than that of those aged 75–99 and 65–74 years, and that this performance was not affected by individuals' sex, number of teeth, occlusal pairs, or dental prosthesis type [25]. Aging‐related syndromes such as sarcopenia, malnutrition, cognitive decline and systemic diseases (e.g., hypertension, obesity and diabetes) may contribute to masticatory dysfunction [36, 37].

In the present study, participants without prostheses or with ill‐fitting ones were still able to perform the chewing gum test, which reflects habitual maximum masticatory performance and captures the chewing ability that naturally occurs with the participant's dental status. This approach aligns with previous studies [6, 11, 25] involving institutionalised older adults. Furthermore, the use of 20 chewing cycles for objective masticatory function follows established protocols for older populations [6, 11, 14, 25] and provides reliable and comparable results across individuals with different dental conditions, while ensuring methodological consistency and reducing variability caused by individual chewing patterns.

Poorer masticatory performance was also associated with a reduced number of natural teeth and POPs. Tooth preservation may play a key role in masticatory performance, but the number of POPs may be a more important parameter, as it may increase chewing efficiency and masticatory performance [22, 36, 38, 39, 40]. To ensure that the inclusion of the variables number of natural teeth and POPs did not introduce bias, potential multicollinearity was assessed. The VIF values ranged from 1.006 to 1.267, indicating that multicollinearity was not a concern, supporting the inclusion of both variables in the adjusted models and reinforcing the validity of the results. Overall, these findings emphasise the importance of oral health maintenance to preserve masticatory function as individuals age.

Masticatory performance was also associated with the need to replace one or more teeth in the upper arch in this study. In a previous study, the lack of a dental prosthesis was associated with poorer masticatory function among partially dentate LTCF residents [9]. The insertion of new dental prostheses is essential to improve older adults' masticatory function and quality of life [41, 42]. These findings reinforce the importance of prosthetic rehabilitation for the restoration of adequate masticatory function among partially dentate older adults, particularly those living in LTCFs.

Poor self‐perceived oral health was associated with poor subjective masticatory function in this study. However, most (63.6%) participants reported good self‐perceived oral health, consistent with previous findings [21]. In other studies, LTCF residents who perceived their oral health to be good had clinically poor oral health [43], likely because they had adjusted their expectations and accepted their limitations [44]. These findings suggest that the use of subjective assessment together with objective measures is important to determine how effectively older people, and especially LTCF residents, can chew and process food.

Subjective masticatory function was associated with the number of POPs in this study. Older adults with reduced numbers of POPs may perceive greater difficulty with chewing due to compromised stability and diminished occlusal efficiency, which is likely to impact their self‐assessments of masticatory ability [45]. Thus, the preservation or restoration of POPs through appropriate prosthetic rehabilitation may contribute not only to improved chewing ability, but also to enhanced OHRQoL, particularly in older populations.

The present study revealed associations of subjective masticatory function with the need for dental prostheses, primarily the need to replace one or more teeth in the upper arch, rather than the need for complete dentures. This apparent contradiction may be explained by several factors. Psychosocial adaptation is one such factor, as partially dentate individuals often report greater dissatisfaction due to both functional and psychological limitations [46]. Evidence indicates that tooth loss is linked to depressive symptoms, mediated by difficulties in chewing, speaking and smiling, thereby highlighting the impact of oral function on overall well‐being [46]. In contrast, completely edentulous individuals may adapt more effectively to their condition [47], particularly when using well‐fitting dentures that provide stable functionality. Additionally, the type of diet commonly offered in LTCFs, typically composed of soft, chopped and well‐cooked foods, may facilitate mastication [47] and, consequently, mitigate the perception of masticatory difficulties across groups. This dietary adaptation could mask functional limitations, especially among completely or partially edentulous LTCF residents. Moreover, individuals requiring tooth replacement may either not be using dentures or may be using ill‐fitting ones [33, 48], which can further influence their perception of impaired function. Unfortunately, the lack of data on denture quality and fit in the present study limits a more comprehensive evaluation of this aspect. These considerations underscore the complexity of interpreting subjective assessments of masticatory function and point to the need for instruments capable of capturing the broader impact of oral status on self‐perceived oral function in institutionalised populations.

In LCTFs, the lack of complete dentures can be normalised, as many older people adapt to edentulism [47]. However, prosthesis quality needs to be assessed in this population, as well‐fitting dentures seem to improve chewing function and oral comfort [49]. Poor denture stability and fit are common issues among LTCF residents [48], exacerbating their chewing difficulties.

Subjective masticatory function was also associated with more severe xerostomia symptoms in this study, in line with the findings of Takagi et al. [16] Xerostomia may occur with hyposalivation or normal salivary flow [30, 50]. Decreased salivary flow can significantly impair masticatory function [38], given the critical roles of normal salivary flow in food breakdown, bolus formation and lubrication, processes that individuals readily perceive. Despite the importance of adequate salivary flow for mastication [36], xerostomia was not associated with objective masticatory function in institutionalised older adults in this study. One possible explanation is that objective parameters may be less sensitive to the perception of xerostomia, partly because chewing gum, used as the test material, stimulates salivary flow in older adults [51] and may temporarily mask symptoms. Additionally, the subjective nature of xerostomia [30] may contribute to these findings, as it can be influenced by psychological factors, functional adaptation, dietary consistency in LTCFs and individual perceptions of discomfort. This may account for its association with subjective rather than objective measures of masticatory function.

The decision to use a single question to assess subjective masticatory function was guided by considerations of simplicity and feasibility in institutionalised older adults, where lengthy or complex instruments may be difficult to apply [52]. Previous research [6], has supported this approach demonstrating its validity as a practical measure of subjective masticatory function. In the present study, the single‐item measure showed consistency with the objective assessment, reinforcing its sensitivity and applicability in this context. Nevertheless, given that subjective perceptions can be influenced by contextual and psychological factors, the use of a single‐item instrument may not fully capture the complexity of masticatory function in all settings, which should be taken into account when interpreting the findings and future studies.

Finally, this study revealed a significant association between objective and subjective masticatory function. This result indicates that individuals with poorer masticatory performance were more likely to report poor subjective masticatory function. Although some clinical factors are associated with objective masticatory function, subjective masticatory function is shaped by individuals' perceptions of their oral health, which are influenced mainly by their psychological condition, cognitive status and general health [16]. Findings from a recent study support the use of qualitative and quantitative assessments, such as the objective and subjective measures employed in this study, to provide deeper insight into the experiences of older LTCF residents [52]. In addition, the use of a single self‐perceived question about chewing may be preferable, as lengthy questionnaires may not add much value [53].

This study has several limitations. Its cross‐sectional design restricted the ability to establish cause‐and‐effect relationships. In addition, the use of a convenience sample can restrict the generalisability of the data and introduce bias. Furthermore, although this study included participants from nine LTCFs, multilevel analysis was not performed. The main reasons were the small number of clusters, the uneven distribution of participants across LTCFs, and the low random effect at the institution level, as indicated by an intraclass correlation coefficient (ICC) below 10%. Therefore, applying hierarchical models would likely yield unreliable estimates. Nevertheless, this limitation should be acknowledged, and future studies with larger numbers of LTCFs are encouraged to consider multilevel approaches to account for potential clustering effects.

Another limitation was that a significant proportion of the initial sample was not included in the final statistical analyses, primarily due to the exclusion of older adults with cognitive impairment or those unable to complete the questionnaires, which is a common challenge in studies involving institutionalised populations. The exclusion of individuals with cognitive decline was necessary because this research was part of a larger study that involved the administration of multiple self‐reported questionnaires, which required sufficient cognitive capacity to ensure the validity and reliability of the self‐reported measures. Although this exclusion was operationally necessary to ensure data reliability, this attrition may have introduced selection bias, limiting the generalisability of the findings to all LTCF residents, given the high prevalence of cognitive impairment in LTCFs. Future longitudinal studies should employ adaptive methodologies to include this vulnerable population, whose participation is essential to generate robust evidence to inform and support care strategies in institutional settings.

Furthermore, data were missing for some variables, including the objective masticatory function, age, self‐perceived oral health, number of POPs, number of teeth and need for a dental prosthesis, a common limitation of observational studies. The condition of dental prostheses was not assessed, which is acknowledged as a limitation. However, prosthetic status was previously examined in another study [11] that specifically addressed this objective.

Although this research was conducted in multiple LTCFs, all institutions were located within the State of São Paulo, which may limit the external validity of the findings. The absence of a multicentre design prevented the inclusion of LTCFs from other regions with potentially different socioeconomic, cultural and institutional characteristics. Local contextual factors, such as institutional infrastructure, number of caregivers per resident, dietary routines and access to health and dental services, may have influenced oral health outcomes and, consequently, the functional perception of older adults. Despite these limitations, the present study contributes novel insights into the distinction and interrelationship between objective and subjective masticatory function among LTCF residents. Future multicentre and longitudinal studies are needed to assess regional and institutional differences and the impact of masticatory function and oral rehabilitation outcomes in this vulnerable population.

## 5 Conclusion

Although the objective and subjective masticatory functions of LTCF residents were associated in this study, related factors differed. The participant age, number of teeth, number of POPs and need for a dental prosthesis in the upper arch were associated with poorer masticatory performance, and poor self‐perceived oral health, the number of POPs, xerostomia and the need for a dental prosthesis in the upper arch were associated with poor subjective masticatory function. Thus, both clinical and self‐perceived factors should be considered when monitoring and addressing masticatory dysfunction in older people living in LTCFs.

## Author Contributions

Mariana Marinho Davino de Medeiros, Yuri Wanderley Cavalcanti and Renata Cunha Matheus Rodrigues Garcia contributed to the conception and design of the study. Lorena Tavares Gama and Mariana Marinho Davino de Medeiros contributed to the acquisition of data. Mariana Marinho Davino de Medeiros performed the data analysis. Lorena Tavares Gama and Mariana Marinho Davino de Medeiros interpreted the data. Lorena Tavares Gama drafted the first version of the manuscript. Mariana Marinho Davino de Medeiros, Yuri Wanderley Cavalcanti, Mario Augusto Brondani and Renata Cunha Matheus Rodrigues Garcia revised the article critically for important intellectual content. All authors read and approved the final version of the manuscript.

## Conflicts of Interest

The authors declare no conflicts of interest.

## Data Availability

The data that support the findings of this study are openly available in Redu/UNICAMP at http://doi.org/10.25824/redu/UX9VV1, reference number UX9VV1.
